# Geriatric Nutrition Risk Index as a predictor of cardiovascular and all-cause mortality in older Americans with diabetes

**DOI:** 10.1186/s13098-023-01060-7

**Published:** 2023-05-02

**Authors:** Xia Shen, Long Yang, Xue Gu, Yuan-Yuan Liu, Lei Jiang

**Affiliations:** 1 Department of Nursing, Wuxi Medical College, Jiangnan University, 1800 Li Hu Avenue, Wuxi, 214062 China; 2 College of Pediatrics, Xinjiang Medical University, Urumqi, China, 393 Xin Yi Road, Urumqi, 830054 China; 3 Department of Radiology, The Convalescent Hospital of East China, No.67 Da Ji Shan, Wuxi, 214065 China

**Keywords:** Geriatric Nutrition Risk Index, GNRI, Cardiovascular mortality, All-cause mortality, Diabetes, Elderly

## Abstract

**Background and aims:**

Few studies have examined the relationship between malnutrition, as defined by the Geriatric Nutrition Risk Index (GNRI), and all-cause mortality and cardiovascular mortality events, particularly in persons with diabetes. The study aimed at the association between GNRI and all-cause mortality and cardiovascular mortality in older Americans with diabetes.

**Methods:**

Data from this retrospective study were obtained from the National Health and Nutrition Examination (NHANES) 1999–2016. Using data from The NHANES Public-Use Linked Mortality Files to assess all-cause mortality (ACM) and cardiovascular mortality (CVM). After excluding participants younger than 60 years and without diabetes, and with missing follow-up data, 4400 cases were left in this study. Persons with diabetes were divided by GNRI into 3 groups: GNRI ≥ 98; 92 ≤ GNRI < 98; and GNRI < 92; (No; Low; Moderate/Severe (M/S) group). We used Cox proportional hazard regression model to explore the predictive role of GNRI on ACM and CVM in elderly persons with diabetes. Restricted cubic splines to investigate the existence of a dose–response linear relationship between them.

**Result:**

During a median follow-up period of 89 months, a total of 538 (12.23%) cardiovascular deaths occurred and 1890 (42.95%) all-cause deaths occurred. Multifactorial COX regression analysis showed all-cause mortality (hazard ratio [HR]: 2.58, 95% CI: 1.672–3.994, *p* < 0.001) and cardiovascular mortality (HR: 2.29, 95% CI: 1.063–4.936, *p* = 0.034) associated with M/S group risk of malnutrition in GNRI compared to no group. A negative association between GNRI and all-cause mortality was observed across gender and ethnicity. However, the same negative association between GNRI and cardiovascular mortality was observed only for males (HR:0.94, 95% CI:0.905–0.974, *p* < 0.001) and other races (HR:0.92, 95% CI:0.861–0.976, *p* = 0.007). And there was no significant correlation between low malnutrition and cardiovascular mortality (*p* = 0.076). Restricted cubic splines showed a nonlinear relationship between GNRI and all-cause mortality and cardiovascular mortality (non-linear p < 0.001, non-linear p = 0.019).

**Conclusions:**

Lower GNRI levels are associated with mortality in older patients with diabetes. GNRI may be a predictor of all-cause mortality and cardiovascular mortality risk in older patients with diabetes.

## Highlight

- The study has a large sample of older diabetes;
- This is the first study of NHANES to explore GNRI and the occurrence of cardiovascular mortality and all-cause mortality.
- We investigated the relationship between GNRI and all-cause mortality and cardiovascular mortality in patients with older diabetes by fitting a COX-restricted cubic spline model.
- Kaplan–Meier curves and log-rank tests were used to assess the probability of survival in diabetes according to GNRI levels.

## Introduction

The prevalence of diabetes has reportedly risen to 8.5% of the global adult population and is estimated by the World Health Organization (WHO) to be the seventh leading cause of death worldwide [1]. And the International Diabetes Federation estimates the number is expected to reach 592 million by 2035 [2]. In addition, people with diabetes are at greatly increased risk for several serious health problems, including macrovascular (cardiovascular disease) and microvascular (retinopathy, nephropathy, and neuropathy) complications [3–5]. Diabetes has been reported to be not only a cause of suffering and a huge financial burden for patients and families but also a significant factor in death and reduced life expectancy in the elderly [6–8].

Malnutrition is likewise a global health problem and is becoming more severe as the global age pyramid changes [9]. In a systematic review, malnutrition diagnosed by nutritional assessment was found to be independently associated with increased ICU length of stay (LOS), ICU readmission rates, the incidence of infection, and in-hospital mortality [10]. Both diabetes and malnutrition affect the subjective quality of life and the incidence of complications as well as life expectancy in hospitalized patients [11, 12]. Patients with diabetes usually are at high risk of malnutrition due to increased nutritional requirements and severe acute inflammatory response [13]. It has been shown that malnutrition is associated with significant modulation of glycemic, hormonal, and cytokine parameters in type 2 diabetes [14]. Therefore, malnourished patients are more likely to have damaged vascular endothelial cells and are more likely to have cardiovascular-related events.

The Geriatric Nutrition Risk Index (GNRI) is an indicator of nutritional status and is a simple and accurate screening tool that includes objective factors such as weight, height, and serum albumin [15]. The ratio of body weight to ideal weight used in GNRI may reflect the degree of debilitation and cachexia associated with poor prognosis in elderly patients [16]. In 2005, Bouillanne and his colleagues first proposed the GNRI as a method to assess the nutritional status of older adults and noted that it could be used to quantify the risk of nutrition-related mortality [17]. To our knowledge, there are no studies assessing the correlation between GNRI and diabetes mortality. Therefore, this study aimed to determine the relationship between GNRI and all-cause mortality and cardiovascular mortality outcomes in elderly patients with diabetes.

## Materials and methods

### Study population

**Fig. 1:**
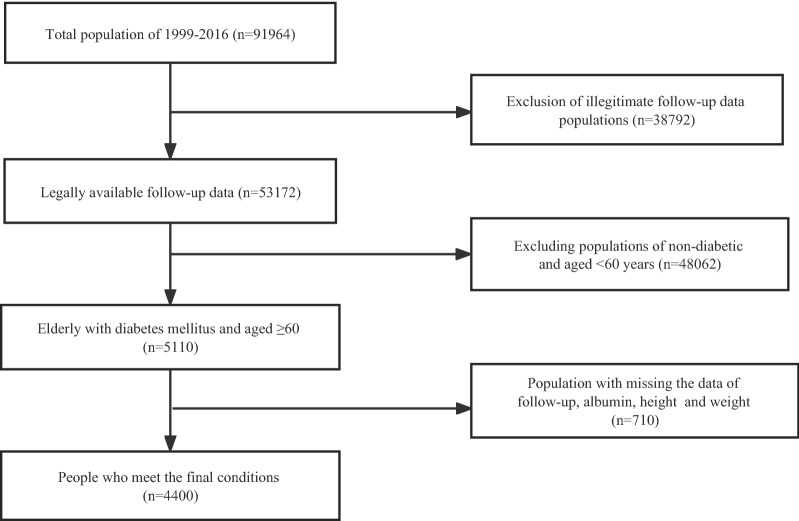
The Flow Chart of Inclusion and Exclusion in the study

Data from nine consecutive National Health and Nutrition Examination Survey (NHANES) cycles from 1999–2016. The NHANES database, which is based on a stratified, multistage, and probability cluster designed and administered by the National Center of Health Statistics of the Centers for Disease Control and Prevention [18]. A mobile examination center (MEC) was used to perform physical examinations and collect blood samples. It includes demographic data, dietary interviews, laboratory tests, and examinations performed by professionally trained staff [19]. In this study, we selected a population with legitimate follow-up data (n = 53,172), excluding persons without diabetes (82%) and subjects younger than 60 years of age (n = 3,096). Further, we excluded data for missing follow-up (4%), albumin (7%), height (3%), and weight (1%), leaving a final sample of 4,400. Additional details of the study sampling and exclusion criteria are shown in Fig. 1. The data were analyzed from November 2022 to January 2023. This study strictly followed the Strengthening the Reporting of Observational Studies in Epidemiology (STROBE) [20]. Furthermore, this study was supported by the National Center for Health Statistics Research Ethics Review Board, and the ethics approval numbers Protocol #98–12, Protocol #2005–06, Continuation of Protocol#2005–06, and Protocol #2011–17. You can find it at this website: NCHS Ethics Review Board Approval (cdc.gov). And all the data used in the manuscript can available on the website: https://wwwn.cdc.gov/nchs/nhanes/search/default.aspx. Informed consent was obtained from each participant.

### Diagnosis of diabetes

The diagnostic criteria for diabetes are including these conditions and must meet one of the points can be: the doctor told you have diabetes, glycohemoglobin (HbA1c) (%) > 6.5, fasting glucose (mmol/L) ≥ 7.0, random blood glucose (mmol/L) ≥ 11.1, two-hour OGTT blood glucose (mmol/L) ≥ 11.1 and use of diabetes medication or insulin.

### The Geriatric Nutrition Risk Index

The Geriatric Nutrition Risk Index (GNRI) was determined by using the following formula: GNRI =  (1.489*serum albumin (g/L)) +  (41.7*body weight (kg)/ideal weight (kg)) [21]. Due to its validity, we calculated the ideal weight using the following formula: 22*square of height [22]. If the patient's weight exceeded the ideal weight, the ratio of weight to ideal weight was set to 1. These variables were used in the baseline examination of the registration. Patients were classified according to the following thresholds [15]: moderate to severe malnutrition risk (M/S risk): < 92; low risk: ≥ 92 to < 98; no risk indicating ≥ 98.

### Covariate assessments

The selection of covariates was based on clinical experience, previous literature [23–27], and the statistical significance of reason. Based on the above, we included the following covariates: Age, gender, Race/ethnicity, BMI, Marital status, Education levels, alcohol consumption, smoking status, serious cardiovascular disease (CVD), Chronic kidney disease (CKD), hypertension, Lymphocyte, Neutrophils, Serum creatinine, Serum uric acid, Triglyceride, Glucose, Glycosylated hemoglobin (HbA1c), Cholesterol, HDL cholesterol, LDL cholesterol, Albumin, Glomerular filtration rate (eGFR), C-reactive protein (CRP), insulin use, Hypoglycemic drugs, Antihypertensive drugs, and Antihyperlipidemic Agents. Race/ethnicity was categorized as Mexican American, non-Hispanic white, non-Hispanic black, or other. Education levels were classified as less than 9th Grade, and higher than 9th Grate or 9th Grate. BMI equals weight (kg) divided by height (m) squared [28]. Marital status was categorized as married, and not married (living with a partner, widowed, divorced, separated, or never married). Participants were categorized as “mild”, “heavy”, and “no” based on the number of drinks per day he/she had drunk. Participants who are “mild” were considered to be drinking alcohol ≤ 1 drink in women and ≤ 2 drinks in men; Participants who are “heavy” were considered to be drinking alcohol ≤ 2 drinks in women and ≤ 3drinks in men or individuals had drunk ≥ 3 drinks of woman and ≥ 4drinks of man; Those who drank before but don't drink now and those who never drank before are defined as “no” [29]. Smoking status was defined as the number and timeline of cigarettes in life (no, smoked less than 100 cigarettes or smoked more than 100 cigarettes in life and smoke not at all now; yes, smoked more than 100 cigarettes in life and smoke some days or every day). CVD consists of coronary heart disease or heart attack or stroke and was assessed by asking participants about their diagnoses. CKD is defined as an estimated eGFR < 60 mL/min/1.73 m^2^ (using the CKD Epidemiology Collaboration equation) and/or a urinary albumin-Cr ratio ≥ 25 mg/g in females and ≥ 17 mg/g in males [30]. Hypertension was collected by questionnaire with a history of hypertension and antihypertensive medication. Blood pressure was measured by a trained physician using a mercury sphygmomanometer with an appropriately sized cuff. And blood pressure measurements were performed three times and the mean of the three measurements was defined as systolic blood pressure (SBP) and diastolic blood pressure (DBP). Hypertension was defined as having a self-reported history of hypertension or use of antihypertensive medication or SBP ≥ 140 mmHg or DPB ≥ 90 mmHg. Other corresponding biochemical data such as Lymphocytes, Neutrophils, Creatinine, serum uric acid, and Triglyceride were obtained from the blood Hemal Biochemistry file. Insulin use is determined by whether or not insulin is used. Hypoglycemic drugs are defined as people who use any of the following blood glucose-lowering drugs: biguanides, sulfonylureas, thiazolidinediones, dipeptidyl peptidase 4 inhibitors, Glp-1receptor agonists, sodium-glucose co-transporter protein 2, alpha-glucosidase inhibitors, or glinides. Antihypertensive drugs are defined as people who use any of the following common blood pressure-lowering drugs: Beta-blockers, Calcium channel blockers, Angiotensin-Converting Enzyme Inhibitors/Angiotensin Receptor Blockers (ACEI/ARB), or diuretics. And statins drugs are defined as Antihyperlipidemic Agents.

### Outcome assessment

To determine the mortality status of the follow-up population, we used the NHANES Public-Use Linked Mortality Files as of December 31, 2019, where National Center for Health Statistics (NCHS) was linked to the National Death Index (NDI) by a probability matching algorithm[8]. Study outcomes included cardiovascular mortality and all-cause mortality, each of which was considered separately. All-cause mortality was defined as death from any cause. Cardiovascular deaths were determined using the International Statistical Classification of Diseases, 10th Revision (ICD-10) codes of I00–I09, I11, I13, and I20–I51 [31]. The median follow-up time of the study was 89 months. All patients were followed until death, loss to follow-up, or study termination date (December 31, 2019).

### Statistical analysis

According to NHANES analysis guidelines, we considered complex sampling designs and sample sizes during data analysis [19]. Sampling weights were calculated as follows: fasting subsample 9-year mobile examination center (MEC) weight = fasting subsample 4-year MEC weight 2/9 (1999–2002) and fasting subsample 2-year MEC weight/9 (2003–2016). And the present data can represent a sample population of 12,400,105. All analyses were performed using the statistical software package R (http://www.r-project.org; version 4.2.2, The R Foundation). Continuous variables were expressed as weighted mean ± standard deviation, and one-way ANOVA was used to compare differences between groups. Categorical variables were expressed as weighted frequencies and percentages and compared using Rao-Scott's χ2 test. A two-sided *p*-value less than 0.05 indicate a denoted statistically significant difference. The Cox proportional hazard regression model was used to calculate the hazard ratios (HR) and 95% (confidence interval) CI for the relationship between GNRI and the prevalence of All-cause and cardiovascular mortality, and the categorical normal group of GNRI (> 98) was used as a reference. For these models, we used untuned and adjusted models. First and foremost, we adjusted for age, gender, CVD, CKD, and Hypertension in Model 1. We further adjusted for education levels, marital status, race/ethnicity, smoking status, alcohol consumption, lymphocyte, neutrophils, Serum creatinine, Serum uric acid, triglyceride, glucose, HbA1c, Cholesterol, HDL cholesterol, LDL cholesterol, eGFR, CRP and the covariates of Model 1 besides (Model 2). Finally, we also adjusted for the variables of whether or not to use insulin, hypoglycemic drugs, antihypertensive drugs, antihyperlipidemic agents, and the covariates of Model 2 (Model 3). We investigated the continuous relationship between GNRI and all-cause mortality and cardiovascular mortality in patients with diabetes by fitting a COX-restricted cubic spline model at the 5th, 35th, 65th, and 95th percentiles of GNRI (22). In addition, Kaplan–Meier curves and log-rank tests were used to assess the probability of survival in persons with diabetes according to GNRI levels.

## Results

### Participant characteristics according to malnutrition risk

In this study, we selected nine continuous NHANES cycles (1999–2000, 2001–2002, 2003–2004,2005–2006, 2007–2008, 2009–2010, 2011–2012, 2013–2014, 2015–2016) and focused on 4,400 diabetes with completed interview and MEC examination in the US (≥ 60 years). Among the 4,400 participants in the study, there were 2,286 males and 2,114 females recruited. Based on the weighted analyses, the mean age of the 4,400 participants was 70.29 years (range, 70.13–70.42 years) and those with an education of above 9th Grate accounted for 25.2%, and most of the participants were non-Hispanic white (41.2%). Participants with higher malnutrition risk were more likely to be female, non-Hispanic black, hypertensive, non-drinkers, and chronic kidney disease patients. For blood biochemistry factors, uric acid, glucose, HbA1c, and CRP were higher in participants who have a low risk of malnutrition. Hypoglycemic drugs use, Antihypertensive drugs use, LDL cholesterol, HDL cholesterol, glucose, serum uric acid, Hypertension, and education levels did not differ in the different malnutrition risks. The baseline characteristics of the participants are summarized in Table 1.

**Table 1:** Baseline characteristics of participants by risk category (GNRI score)

Characteristics	Total	M/S risk (95)	Low risk (416)	No risk (3889)	*p-*value
Demographic information					
Age	70.29 ± 0.13	69.48 ± 0.85	71.56 ± 0.48	70.19 ± 0.14	0.028
Gender					< 0.001
Female	2114 (48)	50 (53.7)	236 (61.0)	1828 (49.4)	
Male	2286 (52)	45 (46.3)	180 (39.0)	2061 (50.6)	
Race/ethnicity					< 0.001
Non-Hispanic black	1026 (23.3)	35 (25.2)	146 (21.4)	845 (11.1)	
Mexican American	888 (20.2)	16 (6.6)	70 (6.4)	802 (6.4)	
Other	675 (15.3)	22 (21.7)	44 (7.9)	609 (10.8)	
Non-Hispanic white	1811 (41.2)	22 (46.5)	156 (64.4)	1633 (71.8)	
BMI	31.31 ± 0.14	32.64 ± 1.37	32.75 ± 0.65	31.15 ± 0.14	0.022
Marital status					0.001
Not married	1968 (44.7)	50 (54.9)	228 (50.1)	1690 (40.1)	
Married	2432 (55.3)	45 (45.1)	188 (49.9)	2199 (59.9)	
Education					0.085
Less than 9th grade	1108 (25.2)	26 (24.9)	101 (15.5)	981 (14.4)	
Higher than 9th grade or 9th grade	3292 (74.8)	69 (75.1)	315 (84.5)	2908 (85.6)	
Alcohol consumption					0.002
Mild	1234 (28)	16 (22.8)	93 (25.7)	1125 (34.5)	
No	2594 (59)	65 (55.7)	278 (66.0)	2251 (53.4)	
Heavy	572 (13)	14 (21.4)	45 (8.3)	513 (12.1)	
Smoking					0.013
No	3879 (88.2)	79 (78.8)	347 (84.5)	3453 (89.6)	
Yes	521 (11.8)	16 (21.2)	69 (15.5)	436 (10.4)	
Chronic Diseases CVD					0.092
No	2941 (66.8)	59 (63.1)	244 (59.3)	2638 (66.0)	
Yes	1459 (33.2)	36 (36.9)	172 (40.7)	1251 (34.0)	
Hypertension					0.308
No	804 (18.3)	14 (11.7)	76 (20.9)	714 (18.4)	
Yes	3596 (81.7)	81 (88.3)	340 (79.1)	3175 (81.6)	
CKD					< 0.001
No	2258 (51.3)	27 (35.0)	158 (38.4)	2073 (54.8)	
Yes	2142 (48.7)	68 (65.0)	258 (61.6)	1816 (45.2)	
Biospecimens					
Lymphocyte (10^^9^/L)	2.03 ± 0.02	1.72 ± 0.12	1.95 ± 0.06	2.04 ± 0.02	0.022
Neutrophils (10^^9^/L)	4.57 ± 0.04	5.22 ± 0.32	4.95 ± 0.11	4.52 ± 0.04	< 0.001
Serum creatinine (umol/L)	93.00 ± 1.04	134.99 ± 14.33	101.27 ± 2.94	91.44 ± 0.98	< 0.001
Serum uric acid (umol/L)	352.08 ± 2.01	365.31 ± 14.83	359.14 ± 7.06	351.15 ± 2.10	0.401
Triglyceride (mmol/L)	2.06 ± 0.03	1.69 ± 0.13	1.99 ± 0.09	2.08 ± 0.03	0.013
Glucose (mmol/L)	8.18 ± 0.06	8.48 ± 0.45	8.50 ± 0.23	8.14 ± 0.07	0.282
HbA1c (mmol/L)	6.91 ± 0.03	7.22 ± 0.21	7.34 ± 0.09	6.86 ± 0.03	< 0.001
Cholesterol (mmol/L)	4.77 ± 0.03	4.53 ± 0.15	4.71 ± 0.07	4.78 ± 0.03	0.134
HDL cholesterol (mmol/L)	1.28 ± 0.01	1.34 ± 0.05	1.30 ± 0.02	1.28 ± 0.01	0.435
LDL cholesterol (mmol/L)	2.55 ± 0.02	2.42 ± 0.10	2.49 ± 0.06	2.55 ± 0.02	0.264
Serum albumin (g/L)	41.67 ± 0.07	31.77 ± 0.40	36.45 ± 0.08	42.36 ± 0.06	< 0.001
eGFR (mL/min/1.73 m^2^)	70.43 ± 0.42	59.54 ± 3.83	65.10 ± 1.62	71.14 ± 0.41	< 0.001
GNRI	103.64 ± 0.10	87.99 ± 0.53	95.61 ± 0.10	104.70 ± 0.10	< 0.001
CRP (mg/dL)	0.46 ± 0.02	1.88 ± 0.35	0.94 ± 0.07	0.39 ± 0.01	< 0.001
Drug use					
Insulin use					< 0.001
No	3712 (84.4)	66 (73.9)	299 (69.8)	3347 (85.6)	
Yes	688 (15.6)	29 (26.1)	117 (30.2)	542 (14.4)	
Hypoglycemic drugs use					0.274
No	1906 (43.3)	47 (52.6)	186 (46.8)	1673 (42.6)	
Yes	2494 (56.7)	48 (47.4)	230 (53.2)	2216 (57.4)	
Antihypertensive drugs use					0.445
No	1021 (23.2)	18 (22.4)	76 (17.9)	927 (21.9)	
Yes	3379 (76.8)	77 (77.6)	340 (82.1)	2962 (78.1)	
Antihyperlipidemic Agents use					0.042
No	474 (10.8)	11 (13.6)	27 (4.0)	436 (8.5)	
Yes	3926 (89.2)	84 (86.4)	389 (96.0)	3453 (91.5)	

M/S risk (moderate/severe risk); BMI (body mass index); Not married (living with a partner, widowed, divorced, separated, or never married); CVD (severe cardiovascular diseases); CKD (chronic kidney disease); HbA1c (Glycosylated hemoglobin); eGFR (Glomerular filtration rate); GNRI (Geriatric Nutrition Risk Index); CRP (C-reactive protein)

M/S risk: GNRI score < 92; low risk: GNRI score ≥ 92 to < 98; no risk: GNRI score ≥ 98

Continuous variables were expressed as weighted mean ± standard deviation, one-way ANOVA was used to compare differences among the different groups. Categorical variables were expressed as weighted frequencies and percentages and compared using Rao-Scott's χ^2^ test

### Association between GNRI and cardiovascular mortality events

**Fig. 2:**
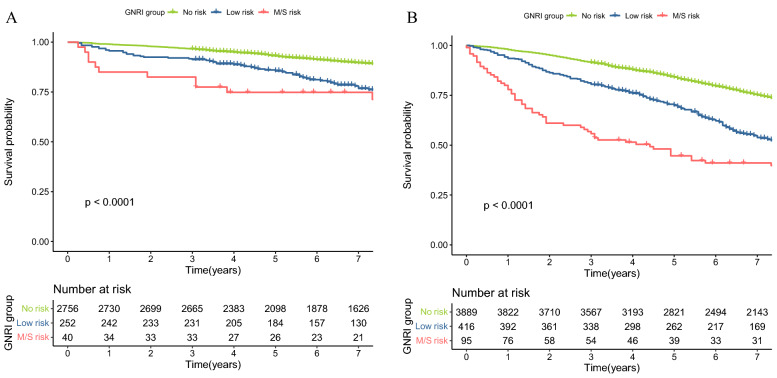
Kaplan–Meier survival rates for mortality among different GNRI groups with older diabetes. (**A**) cardiovascular mortality; (**B**) all-cause mortality

During the follow-up period, a total of 538 (12.23%) cardiovascular mortality occurred in our cohort. Multivariate COX regression analysis showed that each 1-point increase in GNRI was associated with a 5% reduction in the risk of total mortality after adjustment for age, sex, and chronic disease (HR = 0.95, p < 0.001, 95% CI: 0.930–0.978) in Table 2. After correction for laboratory biochemical parameters and medications used, the association between GNRI and the risk of cardiovascular mortality events was still a strong correlation (HR:0.96, *p* = 0.006, [95% CI:0.935–0.989]), and each unit increase in GNRI was associated with a 4% reduction in the risk of death in persons with diabetes. Kaplan–Meier survival rates for cardiovascular mortality differed among no risk, Low risk, and M/S risk (Log-rank *p* < 0.0001), and the survival rates were lowest in the M/S risk group (Fig. 2A). In the crude model of the Cox regression model, the HRs for low risk and M/S risk were 1.94 (1.428, 2.642) and 2.25 (1.201, 4.210), respectively, compared to the no-risk group. After adjusting the models, a significant increase in cardiovascular mortality events were found in the M/S risk group (model 1, model 2, and model 3), where the results after the full model adjusted were (HR:1.55, [95% CI:1.251–1.932]; (HR:2.58, [95% CI:1.672–3.994] in Table 2**.** In the Low-risk group, a significant increase in cardiovascular mortality events compared to the no-risk group was found in model 1, however, the association between GNRI and cardiovascular mortality events was not significant in models 2 and 3, with HRs of 1.403 (0.946, 2.082) and 1.410 (0.965, 2.062), respectively. Furthermore, the results of our subgroup analysis in Table 3 showed a protective effect between GNRI and cardiovascular mortality only among men and other races and interaction between genders (*p* for interaction = 0.006), however, no statistical difference was shown among other races and women.

**Table 2:** Association between GNRI and all-cause and cardiovascular mortality among older Americans with diabetes

Characteristic	Crude model		Model 1		Model 2		Model 3	
	HR (95%CI)	*P*	HR (95%CI)	*P*	HR (95%CI)	*P*	HR (95%CI)	*P*
All-cause mortality								
GNRI	0.945 (0.934,0.955)	< 0.001	0.948 (0.936,0.959)	< 0.001	0.952 (0.941, 0.964)	< 0.001	0.953 (0.942, 0.966)	< 0.001
GNRI (category)								
No risk (n = 3889)	ref		ref		ref		ref	
Low risk (n = 416)	1.832 (1.527,2.199)	< 0.001	1.680 (1.360,2.076)	< 0.001	1.575 (1.266, 1.959)	< 0.001	1.554 (1.251, 1.932)	< 0.001
M/S risk (n = 95)	2.750 (1.930,3.920)	< 0.001	3.101 (2.015,4.772)	< 0.001	2.619 (1.693, 4.052)	< 0.001	2.584 (1.672, 3.994)	< 0.001
*p* for trend		< 0.001		< 0.001		< 0.001		< 0.001
Cardiovascular mortality								
GNRI	0.945 (0.927,0.963)	< 0.001	0.954 (0.930,0.978)	< 0.001	0.961 (0.936, 0.987)	0.004	0.962 (0.935, 0.989)	0.006
GNRI (category)								
No risk (n = 2756)	ref		ref		ref		ref	
Low risk (n = 252)	1.942 (1.428,2.642)	< 0.001	1.583 (1.078,2.324)	0.019	1.403 (0.946, 2.082)	0.092	1.410 (0.965, 2.062)	0.076
M/S risk (n = 40)	2.248 (1.201,4.210)	0.011	2.675 (1.318,5.430)	0.006	2.365 (1.113, 5.024)	0.025	2.291 (1.063, 4.936)	0.034
*p* for trend		< 0.001		0.023		0.123		0.105

Calculated using multivariate COX regression analysis was performed

Crude Model: no adjustment

Model1: Adjusted for age, sex, hypertension, CKD, and CVD

Model2: Adjusted for age, sex, hypertension, CKD and CVD, education level, marital status, race/ethnicity, smoking status, alcohol consumption, lymphocyte, neutrophils, HbA1, glucose, cholesterol, uric acid, creatinine, HDL cholesterol, LDL cholesterol, C-reactive protein, triglyceride, eGFR

Model3: Adjusted for age, sex, hypertension, CKD and CVD, education level, marital status, race/ethnicity, smoking status, alcohol consumption, lymphocyte, neutrophils, HbA1, glucose, cholesterol, uric acid, creatinine, HDL cholesterol, LDL cholesterol, C-reactive protein, triglyceride, eGFR, Antihypertensive drugs, Hypoglycemic drugs, insulin, Antihyperlipidemic Agents

**Table 3:** Association between GNRI and all-cause and cardiovascular mortality in different subgroups

Subgroup	Total	Event (%)	*p*	HR (95% CI)	*p* for interaction
All-cause mortality					
Sex					0.560
Male	2114	828 (49.16)	< 0.001	0.949 (0.934, 0.964)	
Female	2286	1062 (50.84)	< 0.001	0.956 (0.936, 0.976)	
Race/Ethical					0.023
Mexican American	888	309 (4.40)	< 0.001	0.947 (0.920,0.976)	
Non-Hispanic white	1811	987 (76.01)	< 0.001	0.958 (0.943, 0.974)	
Non-Hispanic black	1026	430 (12.08)	< 0.001	0.956 (0.932,0.980)	
Other	675	164 (7.51)	< 0.001	0.916 (0.883,0.950)	
Cardiovascular mortality					
Sex					0.006
Male	1513	227 (46.82)	< 0.001	0.939 (0.905,0.974)	
Female	1535	311 (53.18)	0.517	0.987 (0.947,3.479)	
Race/ethical					
Mexican American	652	73 (3.75)	0.087	0.967 (0.908, 1.030)	0.050
Non-Hispanic white	1121	297 (77.06)	0.117	0.972 (0.937, 1.007)	
Non-Hispanic black	727	131 (12.86)	0.298	0.959 (0.914, 1.006)	
Other	548	37 (6.33)	0.007	0.917 (0.861, 0.976)	

Each stratum was adjusted for age, hypertension, CKD and CVD, education level, marital status, race/ethnicity, smoking status, alcohol consumption, lymphocyte, neutrophils, HbA1, glucose, cholesterol, uric acid, creatinine, HDL cholesterol, LDL cholesterol, C-reactive protein, triglyceride, eGFR, Antihypertensive drugs, Hypoglycemic drugs, insulin, Antihyperlipidemic Agents

### Association between GNRI and All‑cause mortality

During the follow-up period, a total of 1890 (42.95%) patients died. Multifactorial COX regression analysis showed a strong correlation between GNRI and risk of all-cause mortality even after adjustment for the full model (HR:0.95, [95% CI:0.942–0.966], *p* < 0.001). there was a significant difference in Kaplan–Meier survival rates for all-cause mortality among the no-risk, low-risk, and M/S risk groups (*p* < 0.001) (Fig. 2B), while the M/S risk group had the lowest survival rate. All-cause mortality was significantly increased in the low-risk and M/S risk groups compared to the no-risk group in the crude, all-multivariate model (Table 2). In addition, we found a negative association between GNRI and all-cause mortality both by gender and race in the corresponding subgroup analysis, and there is an interaction between races (*p* for interaction = 0.023) in Table 3.

### Dose–response relationship between cardiovascular mortality and GNRI in older diabetes

**Fig. 3:**
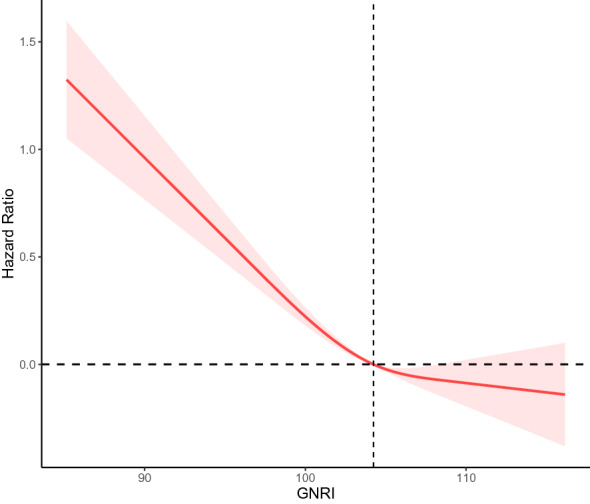
Dose–response relationship between cardiovascular mortality and GNRI in older diabetes Adjusted for age, sex, race/ethnicity, education levels, marital status, smoking status, alcohol intake, hypertension, CVD, CKD, lymphocyte, neutrophils, Serum creatinine, Serum uric acid, triglyceride, glucose, HbA1c, Cholesterol, HDL cholesterol, LDL cholesterol, eGFR, CRP, insulin use, hypoglycemic drugs, antihypertensive drugs, antihyperlipidemic agents

In a restricted cubic spline regression analysis model fully adjusted for confounders, we observed an L-shaped association between GNRI and cardiovascular mortality in elderly persons with diabetes (non-linear p = 0.019). With increasing GNRI, there was a trend toward progressively lower and then higher cardiovascular mortality events, as shown in Fig. 3**.**

### Non-linear correlation analysis of All‑cause mortality and GNRI in older diabetes

**Fig. 4:**
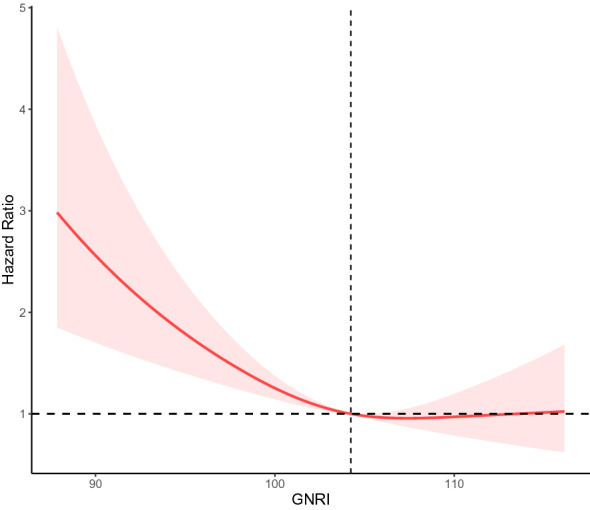
Non-linear correlation analysis of All‑cause mortality and GNRI in older diabetes. Adjusted for age, sex, race/ethnicity, education levels, marital status, smoking status, alcohol intake, hypertension, CVD, CKD, lymphocyte, neutrophils, Serum creatinine, Serum uric acid, triglyceride, glucose, HbA1c, Cholesterol, HDL cholesterol, LDL cholesterol, eGFR, CRP, insulin use, hypoglycemic drugs, antihypertensive drugs, antihyperlipidemic agents

Dose–response curves between GNRI and the risk of all-cause mortality showed a nonlinear negative association between GNRI and the risk of all-cause mortality in elderly persons with diabetes (non-linear *p* < 0.001) in Fig. 4. The risk of all-cause mortality decreased progressively with increasing GNRI values, especially when GNRI values were less than 102.75.

## Discussion

In this study, we revealed two important findings. First, increasing nutritional risks such as low risk, and M/S risk of GNRI, could be a new predictor of all-cause mortality in elderly patients with diabetes. Second, the M/S risk of GNRI scores was associated with cardiovascular mortality events. These results suggest that malnutrition may be a potentially modifiable risk factor for reducing the risk of death in elderly patients with diabetes mellitus.

### GNRI as a predictor of mortality risk

GNRI is an indicator of the nutritional status of the elderly. It is calculated using serum albumin levels, weight, and height. In addition, it involves a dual assessment of serum albumin and BMI, which in turn complements and improves its diagnostic accuracy. Yamada [32] et al. used GNRI to evaluate the nutritional status of hemodialysis patients and stated that GNRI is the most sensitive, specific, accurate, simple, and objective evaluation method among the five nutritional screening tools. GNRI has high reproducibility and no observational bias compared to other subjective evaluation methods. And it has been confirmed that the predictive value of GNRI for nutritional risk and its correlation with prognosis is higher than the univariate of albumin or BMI [15, 33]. In addition, GNRI can be used to screen people at high nutritional risk by operating the computer, which facilitates long-term, regular, and large sample monitoring and follow-up [17].

Nutritional status has been reported to be an important predictor of mortality from various diseases [17, 34–36]. Long-term chronic diseases, including diabetes, can lead to malnutrition, which may exacerbate disease progression and lead to a poor prognosis [37, 38]. Recently, many studies have now demonstrated the value of GNRI in assessing the nutritional status and predicting the prognosis of patients with hemodialysis disease in Asia [33, 39]. A retrospective cohort study from the First Hospital of Wenzhou Medical University suggested that GNRI could be an independent prognostic indicator for patients with severe diabetic foot ulcers [40]. When tools other than GNRI are used to assess patient mortality risk, the results continue to support our study. It has been suggested that a low GNRI is not only a strong predictor of all-cause mortality in patients with chronic kidney disease but is also highly associated with the risk of cardiovascular events [41]. Similarly, Yong mei. et al. [42] noted that serum cholesterol levels in malnourished patients were negatively associated with all-cause mortality and cardiovascular mortality. To our knowledge, there are no studies that have separately explored the relationship between GNRI and its mortality in persons with diabetes. Our study says GNRI can be a fine predictor of prognosis in older adults with diabetes.

Serum albumin is considered a clinical monitoring tool for nutritional assessment, and hypoalbuminemia has been shown to be strongly associated with complications and mortality in the elderly [43]. Therefore, hypoalbuminemia is considered a predictive risk factor for mortality. Furthermore, albumin is an important factor in the GNRI equation and therefore may potentially explain the relationship between GNRI and mortality in persons with diabetes. The increased morbidity and mortality associated with diabetes may be explained because they present with complications affecting almost all body organs, for example, well-characterized macrovascular and microvascular complications include cardiovascular disease (CVD), retinopathy, neuropathy, and chronic kidney disease [44]. At the same time, increasingly diverse and non-vascular diabetes complications are becoming common, including psychiatric disorders (depression), cancer, cognitive impairment, infection, and disability [45]. In a 2018 Australian study that surveyed 700,000 adults from the Australian National Register of People with Type 2 Diabetes, all-cause mortality, CVD mortality, stroke, and ischemic heart disease mortality were greatly increased in people with diabetes [46].

Noticeably, GNRI showed an L-shaped association with all-cause mortality in the diabetes population. A linear curve with a GNRI range of 100 to 110 became stable, with an increasing trend above 110. This suggests that GNRI may become a risk factor for cardiovascular disease when it exceeds a certain range. This may be explained by obesity or overnutrition as a risk factor for CVD [47, 48].

### Pathophysiological association between GNRI and mortality in elderly persons with diabetes

Firstly, malnutrition increases the risk of infection [49]. Both lymphocyte function and innate host defense mechanisms (macrophages and granulocytes) are affected [50]. Second, malnutrition not only promotes acute and chronic infections, but also leads to increased food intake, nutrient absorption, direct or catabolic nutrient losses, and metabolic demands [13, 51]. Under inflammatory conditions, mediators increase the catabolic disease state, characterized by enhanced arginine use. This depletion of amino acids impairs the T-cell response [52], and once the body exceeds arginine production, the body leads to a negative nitrogen balance [53]. Similarly, it has been reported that inhibiting the expression of inflammatory cytokines and chemokines can reduce cardiovascular risk in mice [54]. In addition, malnutrition leads to immunosuppression through several mechanisms, including the involvement of leptin and the hypothalamic–pituitary–adrenal axis. On the other hand, Protein energy malnutrition decreases leptin concentrations and increases serum levels of the stress hormone glucocorticoid [55, 56]. Therefore, it is likely that the hypothalamic–pituitary–adrenal axis plays a key role in malnutrition-related immune defense. It has been suggested that the function of autophagosomes and adrenergic receptors plays an important role in ventricular remodeling in mice with diabetic cardiomyopathy [57]. In contrast, diabetes is a metabolic disease regulated by hormones that affect the normal function of the immune system [51]. The function of neutrophils and macrophages is suppressed in persons with diabetes, including phagocytosis, production of reactive oxygen intermediates, chemotaxis, and extravasation. Activation of T cells is also compromised, and the production of reactive oxygen intermediates requires reductive coenzyme II, which is consumed by the gluconeogenic pathway [58]. As a result, persons with diabetes are more prone to complications and therefore have a higher mortality and morbidity rate of diabetic malnutrition.

## Advantages and limitations

Our study has several advantages. First, our study is the first to show an association between GNRI levels and mortality in a longitudinal cohort study of a large number of persons with diabetes. Second, we explored the relationship between GNRI and cardiovascular mortality and all-cause mortality, respectively. In addition, we adjusted for as many confounding factors as possible, so the results may be more convincing. There are also several limitations to this study. First and foremost, despite our rigorous adjustment for baseline clinical characteristics, our observations may be influenced by unmeasured and unknown confounders. Furthermore, because the NHANES study collected data at one point in time, nutritional data such as serum albumin, height, and weight were recorded only once for all participants, which may lead to bias in GNRI calculations.

## Conclusions

This study confirms that lower GNRI scores are highly associated with the risk of all-cause mortality and cardiovascular mortality in persons with diabetes. To avoid premature death among adults with diabetes in the United States, it is recommended that they focus on a balanced nutritional intake in their daily lives. Clinical care workers should also pay attention to assessing the nutritional status of patients and give them timely and appropriate dietary guidance. This study provides a significant reference for reducing premature mortality in the diabetic population with adequate nutritional intake as a primary prevention strategy!

## Data Availability

All the data are available to the public and were used in the manuscript can available on the website: https://wwwn.cdc.gov/nchs/nhanes/search/default.aspx
